# Complementary and Alternative Medicine Use among Physicians in Oriental Medicine Hospitals in Vietnam: A Hospital-Based Survey

**DOI:** 10.1155/2013/392191

**Published:** 2013-03-24

**Authors:** Duong Duc Pham, Jong Hyang Yoo, Binh Quoc Tran, Thuy Thu Ta

**Affiliations:** ^1^Korea Institute of Oriental Medicine, 461-24 Jeonmin-dong, Yuseong-gu, Daejeon 305-811, Republic of Korea; ^2^National Hospital of Traditional Medicine, 29 Nguyen Binh Khiem, Hanoi 112611, Vietnam

## Abstract

Interest in complementary and alternative medicine (CAM) is growing worldwide, even in Vietnam where traditional medicine is considered mainstream. We conducted a survey of the knowledge, attitudes, and practices of CAM therapies among physicians in oriental medicine (OM) hospitals in Vietnam. A two-stage random selection process selected 337 physicians who were interviewed using a face-to-face method with a standardized structured questionnaire. Data from 312 physicians who completed the questionnaire suggested that oriental herbal medicine and acupuncture (Vietnamese OM version) were the more commonly used CAM modalities compared with Vietnamese folk medicine and other forms of CAM. A broad range of CAM modalities, particularly chiropractice, diet supplements, and dietary therapy, and an excessive proportion of western medication were employed in conjunction with OM in the physicians' daily practice. Their daily practice was influenced by the source of knowledge, education level, medical specialty, and working environment. These findings suggest that physicians in OM hospitals in Vietnam have interests in various forms of CAM therapies besides traditional modes.

## 1. Introduction

Complementary and alternative medicine (CAM) refers to a broad range of health care practices and products that are not considered part of allopathic medicine but are considered a supplement [1]. In some countries where the traditional medical knowledge, skills, beliefs, and experiences have been widely accepted and integrated into the mainstream health care system, CAM can be interchangeable with traditional medicine (TM) [2]. TM practiced in the Western Pacific Region, including traditional Chinese medicine (TCM), traditional Korean medicine, Kampo medicine, and traditional Vietnamese medicine (TVM), were more or less influenced by the ancient holistic medical system from China, and these TMs have been considered using the common term “oriental medicine” (OM) [3].

In recent decades, there has been a remarkable growth in CAM practice worldwide, both in the west and in the east. In the USA, 38.3% of adults and 11.8% of children have used some forms of CAM [4], whereas a large number of physicians in Europe and North America have referred their patients for acupuncture (43%), chiropractice (40%), and massage (21%) [5]. In Korea, demand for OM has increased rapidly, with 57 OM hospitals and 4723 OM clinics newly established from 1996 to 2007 [6]. In regional surveys, 73% of Japanese medical doctors practiced CAM in 1999 [7], and that percentage increased to 80% in 2005 [8]. However, the commonly used modalities of CAM have varied between countries and over time. In the west, CAM users and providers tend to use a wide range of CAM therapies, including natural products, deep breathing, chiropractice, meditation, Ayurveda, and homeopathy [4, 9], whereas OM has been the most frequently used CAM modality in the east because it was officially included in the medical college curriculum [7, 8, 10]. The globalization and academic exchange of OM practitioners in the east have allowed practitioners to become more familiar with other CAM therapies and employ these treatments in their daily practice [8, 10], while the CAM practitioners in the west tended to integrate OM into their expertise [11].

TVM includes two aspects: the enriched identity of indigenous medicine characterized by a huge collection of healthcare experience using native herbs and animals, that is, the so-called Vietnamese folk medicine; the Vietnamese version of OM [12], which has been integrated into the mainstream health care system in Vietnam for the last 50 years. In Vietnam, during the 6-year medical school program, general practitioners (GP) and OM doctors (OMD) complete the same courses on basic medical science within the first four years before focusing on allopathic medicine and TVM, respectively. However, GPs are capable of TVM practice if they take graduate courses on TVM and pass their examinations, whereas OMDs are allowed to employ both TVM and allopathic medicine immediately after graduation. In Vietnam, almost all of the TVM components such as herbal medicine, acupuncture, acupressure, and massage are covered by the national health insurance, whereas other modalities of CAM are paid by out-of-pocket expenditure. Although TVM has been considered the main CAM modality in Vietnam, there has been an increase in the interest and practice of other CAM therapies concurrent with economic growth and growing international exchange. However, no previous studies have examined this trend.

To date in Vietnam, national and provincial OM hospitals have contributed as the main part of the national system for OM. These OM hospitals provide not only high quality OM services but also technical guidance of OM to the community. We therefore surveyed the knowledge, attitude, and practical use of CAM therapies among the physicians who work in OM hospitals to understand the current trends in CAM use in Vietnam and the factors that attribute to the trends.

## 2. Methods

### 2.1. Sample

**Figure 1:**
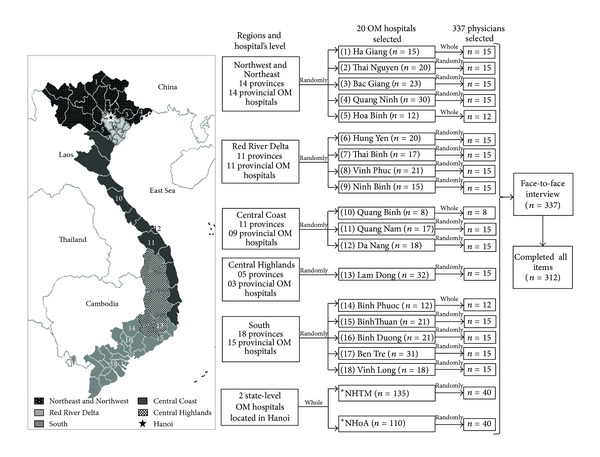
Flow chart of sample selection. The provinces of Vietnam were divided into 5 regions, including the Northeast and Northwest, the Red River Delta, Central Coast, Central Highlands, and Southern regions. (1) The OM hospital of Ha Giang province; (2) the OM hospital of Thai Nguyen province; (3) the OM hospital of Bac Giang province; (4) the OM hospital of Quang Ninh province; (5) the OM hospital of Hoa Binh province; (6) the OM hospital of Hung Yen province; (7) the OM hospital of Thai Binh province; (8) the OM hospital of Vinh Phuc province; (9) the OM hospital of Ninh Binh province; (10) the OM hospital of Quang Binh province; (11) the OM hospital of Quang Nam province; (12) the OM hospital of Da Nang city; (13) the OM hospital of Lam Dong province; (14) the OM hospital of Binh Phuoc province; (15) the OM hospital of Binh Thuan province; (16) the OM hospital of Binh Duong province; (17) the OM hospital of Ben Tre province; (18) the OM hospital of Vinh Long province. The star is Hanoi, the capital of Vietnam, where there are two state-level OM hospitals: NHTM: the National Hospital of Traditional Medicine and NHoA: the National Hospital of Acupuncture.

Physicians who work in national and provincial OM hospitals and who directly provide treatment to patients were recruited for the survey using a two-stage random selection. Radiologists, clinical pathologists, anesthesiologists, and those who rarely independently see patients were excluded. At the time that we conducted the survey (from September to October 2012), there were 2 national OM hospitals and 52 provincial hospitals in the national system for OM in Vietnam. This study randomly selected 18 provincial OM hospitals based on an estimated ratio of the 5 geographic regions, whereas the National Hospital of Traditional Medicine and the National Hospital of Acupuncture, both state-level OM hospitals, were included. In the second stage, 40 physicians from each national OM hospital and 15 physicians from each provincial OM hospital were selected randomly. If the total number of physicians in a certain hospital was not over 15, then all of the physicians were enrolled. Face-to-face interviews were conducted for 337 selected participants, and those who did not complete all of the questionnaire items were excluded. Therefore, data from 312 participants were used for analysis (Figure 1). 

### 2.2. Data Collection

The interviews were performed by trained interviewers who followed a standardized protocol and used a structured questionnaire. Demographic information of the participants, including their age, gender, department in which they worked (inpatient or outpatient wards), education in TM (undergraduate or graduate), length of experience in TM practice, experience running a private clinic, type of specialty training received in medical school (GP or OMD), and the number of patients seen per day, was collected. At first, the participants were asked whether they were familiar with the concept of CAM and whether TVM belongs in the category of CAM. Then, a definition of CAM defined as any form of medicine that does not belong to modern western medicine and that is used equally and along with TM, a familiar concept to Vietnamese OMDs, was provided to the interviewees and was used consistently in each question. The participants were asked to self-assess the degree of their knowledge based on four possible answers (3 = I know well enough for clinical practice, 2 = I know but not well enough for clinical practice, 1 = I have heard, and 0 = I have never heard) of twelve CAM therapies, including oriental herbal medicine, oriental acupuncture, Vietnamese folk medicine, dietary supplements, Qigong, chiropractice, yoga, dietary therapy, reflexology, aromatherapy, Ayurveda, and homeopathy. The questionnaire also asked whether the participants' knowledge of CAM originated from their medical school curriculum, textbooks, training and academic conferences, academic journals, general newspapers and magazines, inherited knowledge from other OM practitioners in the community, and inherited knowledge from their relatives. For the questions about CAM practice, the participants were asked to identify using a single-choice method (1 = Yes, 0 = No) which of the twelve aforementioned CAM therapies that they have ever used in their daily practice. 

The attitude toward CAM therapies was assessed by questions regarding the participant's belief in the substantial effectiveness, holistic approach, natural source, and superiority of CAM therapies compared with conventional medicine. The participants were also asked whether CAM use should be based on scientific evidence. The answers for the attitude items were rated on a four-point scale (3 = absolutely agree, 2 = agree, 1 = disagree, and 0 = absolutely disagree). 

This study was approved by the IRB of the National Hospitals of Traditional Medicine of Vietnam.

### 2.3. Data Analysis

The overall scores of knowledge (from 0 to 36 points) and practice (from 0 to 12 points) were calculated as the sum of the knowledge scores and practice scores of the 12 items, respectively. Cronbach's *α* coefficients for the knowledge and practice items were 0.84 and 0.70, respectively. The corrected item-total correlation coefficients for knowledge items were between 0.36 and 0.63, whereas these values for practice items were between 0.22 and 0.47, except for homeopathy (0.03) and Ayurveda (0.01) those which are not familiar CAM modalities in Vietnam. However, the Cronbach's *α* coefficient for practice items (0.704) did not change significantly if these two items were deleted (0.708 and 0.709, resp.). We therefore included homeopathy and Ayurveda items in calculating practice overall score. A high overall score of knowledge and practice of CAM indicated an abundance of knowledge of CAM and the diversity of CAM use in clinical practice. The overall score of attitude toward CAM was calculated as the sum of scores across the four attitude items (Cronbach's *α* coefficient was 0.57, and the corrected item-total correlation coefficients were between 0.30 and 0.42). The fifth attitudinal item, which surveyed the need of evidence-based CAM, was assessed separately because it reflected a different aspect. A high overall attitudinal score indicated a positive attitude toward CAM. Chi-squared tests were used for categorical variables. To assess the association between the demographic characteristics, the knowledge, practice, and attitude scores and the proportion of western medication use, multiple linear regression analysis was employed. Age, gender, department, number of patients seen per day, length of experience in TM practice, education level, experience running a private clinic, type of specialty training received in medical school, and hospital level were considered as predictive factors. The data were analyzed using SPSS 19.0 (SPSS Inc, Chicago, IL), and the significance level was set to 0.05.

## 3. Results

### 3.1. Response Rate

A total of 312 physicians completed all the items of the questionnaire (92.6%), and the response rates of those who worked in provincial hospitals (93.0%) and those who worked in national hospitals (91.3%) were comparable. 

### 3.2. Demographic Characteristics of Participants

The respondents included 156 men and 156 women with ages ranging from 22 to 60; 237 (76.0%) physicians worked in inpatient wards, 147 (47.1%) physicians had graduate degree in TM, and 87 (27.9%) physicians were trained as GPs in medical school. A few physicians (24.7%) were running private clinics after working at the hospitals. 

The physicians in the national hospitals were older, had more education, and were more experienced than those in the provincial hospitals. There was a higher proportion of participants who were trained as GPs in medical school (38.4% versus 24.7%) and who worked in outpatient wards (35.6% versus 24.7%) in the national hospitals compared with those in provincial hospitals. However, the number of patients seen per day by the physicians in the provincial hospitals was significantly higher than that seen by those in the national hospitals. There were no differences in gender and private clinic ownership between the respondents from the national and provincial hospitals (Table 1).

### 3.3. Knowledge, Practice, and Attitude towards CAM

**Figure 2:**
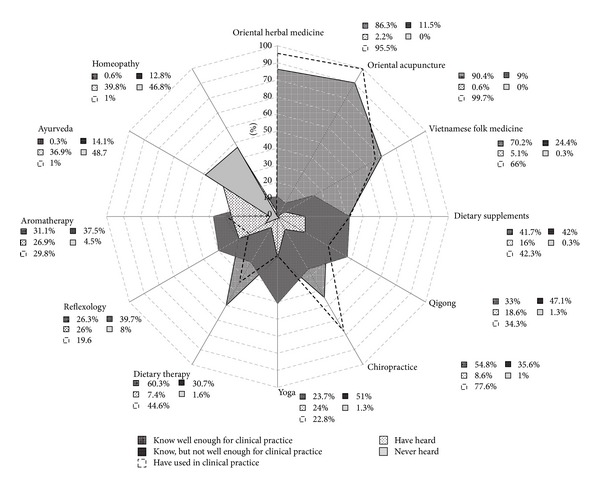
Knowledge and practice of CAM therapies among physicians in oriental medicine hospitals. This spider web chart represents proportion of positive response to four scale 12-item questionnaire about knowledge of CAM modalities versus proportion of CAM modalities used in clinical practice. Each axis indicates the proportion of positive response from 0 to 100% for a given CAM modality.

A large proportion of the physicians (80.8%) had heard of the concept of CAM; among them, 188 physicians (74.6%) regarded TVM as a form of CAM (data not shown). Oriental herbal medicine and acupuncture, the main components of OM, were practiced by almost all of the physicians in the OM hospitals (95.5% and 99.7%, resp.) and were well known by almost all of the respondents (86.2% and 90.4%, resp.). The second most popular group of CAM therapies included Vietnamese folk medicine, chiropractice, and dietary therapy, with a relatively high proportion of physicians (between 54.8% and 70.2%) who had sufficient knowledge of and who practiced these CAM therapies (between 44.6% and 77.6%). Some physicians in the OM hospitals had sufficient knowledge of and had practiced with dietary supplements (41.7% and 42.3%, resp.), Qigong (33.0% and 34.3%, resp.), aromatherapy (31.1% and 29.8%, resp.), and reflexology (26.3% and 19.6%, resp.), whereas very few doctors had sufficient knowledge of and had practiced homeopathy (0.6% and 1.0%, resp.) and Ayurveda (0.3% and 1.0%, resp.) (Figure 2). 

Because the OMDs in Vietnam can prescribe both western medication and OM, we surveyed the average proportion of western medication use in combination with OM in daily practice. Almost all of the physicians in the OM (99.7%) reported combining western medication with OM in their daily practice, and the average proportion of combination was ranging from 5% to 100%, with mean and standard deviation of 42.4% and 26.5%, respectively, and a median of 30% (data not shown).

A higher proportion of physicians had knowledge of CAM that was obtained from their medical school curriculums (98.7%), textbooks (84.0%), academic journals (77.2%), the internet (73.7%), and conference/training (72.8%), whereas a few doctors had inherited their CAM knowledge from practitioners in their family and in the community (28.8% and 27.9%, resp.). The proportions of physicians in the provincial and national OM hospitals who had CAM knowledge that originated from official sources such as medical school and textbooks were comparable, whereas the proportion of those who received CAM knowledge from academic journals, conference/training, the internet, general newspapers, and magazines or inherited the knowledge from CAM practitioners in the community among the physicians in the national OM hospitals was higher than the proportion of the respondents in provincial hospitals (Table 2).

As shown in Table 3, a high proportion of physicians in OM hospitals believed in the true effectiveness (100%), the advantages of their natural sources (93.9%), holistic approach (75%), and the superiority (96.2%) of CAM therapies. However, approximately 80% of the physicians agreed that CAM use should be based on scientific evidence.

### 3.4. Factors Influence Knowledge, Practice, and Attitude towards CAM

The overall score of attitude toward CAM was weakly correlated with the overall scores of knowledge (*r* = 0.27, *P* < 0.0001) and practice (*r* = 0.28, *P* < 0.0001) of CAM, whereas there was a high correlation between overall knowledge and practice scores of CAM (*r* = 55, *P* < 0.0001) (data not shown).

The regression analysis showed that age, gender, department (inpatient or outpatient wards), number of patients seen per day, and running a private clinic were not associated with the overall scores of knowledge, practice, and attitude toward CAM and the proportion of western medication use. Physicians who had a graduate education in TM tended to have a higher overall score of knowledge (26.5 versus 23.7, *P* < 0.001), practice (6.3 versus 5.1, *P* < 0.001), and attitude (10.5 versus 9.7, *P* < 0.01) toward CAM compared with those who had only a bachelor's degree. The respondents who had graduated as OMDs tended to have a higher overall knowledge score of CAM (25.8 versus 24.4, *P* < 0.05) and a lower average percent of western medication use (41.8 versus 55.0, *P* < 0.001) compared with those had graduated as GPs. Participants in the national OM hospitals were more likely to have a higher overall score of knowledge (26.5 versus 23.7, *P* < 0.001), practice (6.2 versus 5.2, *P* < 0.01) of CAM, and a higher average proportion of western medication use (54.5 versus 42.3, *P* < 0.01) compared with those who worked in the provincial OM hospitals. Physicians who had less than 10 years of TM practice tended to have a higher overall score of knowledge (26.6 versus 23.3, *P* < 0.01) and attitude (10.5 versus 9.5, *P* < 0.01) than those who had more than 20 years of TM practice (Table 4). Almost all aforementioned statistical significances remained with Bonferroni correction, except for the association between medical specialty and knowledge score (99.4% confident interval were between −0.26 and 3.22) (data not shown).

## 4. Discussion

TVM is composed of two parts: the Vietnamese version of OM and Vietnamese folk medicine. TVM has been widely used in Vietnam as an integral part of the national mainstream health care system. Because of globalization, various other forms of CAM have been adopted and have spread among Vietnamese CAM practitioners. This study is the first attempt to investigate the current trends in CAM practice, including TVM in OM hospitals, which provide the most CAM services in Vietnam, by assessing the knowledge, practice, and attitude toward CAM among physicians in the OM hospitals.

This study showed that oriental herbal medicine and acupuncture, the main components of OM, were most well known and practiced by the physicians in OM hospitals and were more commonly used than Vietnamese folk medicine, the other component of TVM. In addition to TVM, many physicians have employed other CAM modalities in their daily practice, including chiropractice (77.6%), dietary therapy (44.6%), dietary supplements (42.3%), Qigong (34.3%), aromatherapy (29.8%), yoga (22.8%), and reflexology (19.6%), whereas the physicians rarely practiced Ayurveda and homeopathy. Another characteristic of CAM practice among the physicians in OM hospitals was a high proportion of western medication use that was integrated with OM (42,4%), even among OMDs (41.8%).

In accord with findings from Japan and Korea, OM has been the most commonly used CAM modality by OMDs and physicians [7, 8, 10]. The main reason of this trend may be that OM originated from ancient Chinese medicine that was introduced to Japan, Korea, and Vietnam a thousand years ago and is considered part of the oriental culture [3]. The other reasons may be that OM courses have been provided in medical school, even for GPs in Japan and Vietnam [2, 7], and that many OM services are covered by the national health insurance systems in these countries [2]. However, acupuncture has not been widely used by Japanese physicians (8%), although it is the most popular CAM therapy in Korea and Vietnam [8, 10]. Vietnamese OMDs are officially authorized to prescribe western medication, whereas the OMDs in Korea and Japan can only refer patients to western medical doctors for conventional interventions. The intense combination of western medication with CAM practice may encourage the practice of integrative medicine by OMDs, but western medication that has been abused by OMDs is unknown. Further investigation should be conducted on how, when and where the integration of western medication in CAM practice is performed. Other forms of CAM such as chiropractice, dietary supplement, and aromatherapy are also known to and practiced by a large number of GPs and OMDs in these Asian countries, but Ayurveda and homeopathy were rarely used [8, 10]. The trend in which OM contributes as the mainstream modality of CAM use, in addition to the increasing interest regarding other CAM modalities, is in contrast with the trend in American and European countries, where no particular CAM modality dominates in popularity [4, 9]. Burg et al. found that the most commonly used CAM therapies among health professionals in Florida were massage (32%), dietary supplements (28%), and relaxation techniques (24%) [13], whereas Kurtz et al. reported a heterogeneity in the CAM therapies used by primary care physicians in Michigan, which included vitamin therapy (32.4%), herbal therapy (20.8%), mineral therapy (19.6%), and dietary therapy (19.6%) [11].

Although the physicians in OM hospitals in Vietnam have a strong favorable attitude toward CAM, a large proportion of them also desired to practice CAM based on scientific evidence. Solid evidence and reliable information are influential factors for the attitude and practice of CAM therapies among medical doctors [14, 15]. Further study in the future should be conducted on the evidence-based CAM practice in Vietnam. 

Various factors may influence CAM use. The dominance of OM and disregard for folk medicine in the medical school curriculum may be attributed to the higher use rate of oriental herbal medicine (95.5%) and acupuncture (99.7%) rather than Vietnamese folk medicine (66.0%). Another reason for this difference may be that few physicians (less than 30%) reported having inherited their CAM knowledge from practitioners in the community or in their family who practiced folk medicine rather than OM. On the other hand, the out-of-school knowledge, particularly from the internet (73.7%), may be implicated in the heterogeneity of the CAM modalities used by the physicians, including chiropractice, dietary therapy, and dietary supplements.

A previous study suggested that younger physicians used more CAM therapies in their daily practice [10, 16]. In this study, the abundance of knowledge of and the favorable attitude toward CAM were not related to age, but the length of TM practice. Interestingly, the respondents with fewer years of TM practice had more knowledge and a more favorable attitude. A possible explanation may be that younger physicians are familiar with a broader range of CAM modalities rather than focusing only on TVM, which is the tendency of those who have a large amount of experience in TM practice. Those who practice TM for a long period of time may have a reasonable belief in the effectiveness of CAM and TM, whereas beginners tend to overestimate the effectiveness of CAM therapies. However, we found no difference in the heterogeneity of CAM use by age and length of TM practice. 

Education level in TM, hospital level, and specialty of doctor (GP or OMD) appeared to be crucial influential factors for the trend of CAM use among physicians in OM hospitals in Vietnam. We found that physicians who had graduate education degrees and who worked in national OM hospitals possessed a broader knowledge of CAM therapies along with the application of various CAM modalities, regardless of other demographic factors. Although OM has been the main content included in graduate courses in OM medical schools, graduate students may have a chance to expand their knowledge of various CAM modalities during the course, and then they may integrate different CAM therapies in their practice. This finding suggests that further education in TM brings about more benefits for physicians and should be encouraged. The working environment may also influence the CAM use of physicians. The physicians in the national OM hospitals may have a better chance of learning from outstanding CAM or OM experts, and they have access to a large resource of academic references (e.g., journals, conference, and training). We also found that the knowledge sources of CAM therapies were different by working environment. The physicians in the national OM hospitals had more opportunities to gain out-of-school knowledge on CAM (e.g., from academic journals, the internet, conference/training, and magazines) than those in lower level hospitals (Table 2).

This study also underlined the excessive use of western medication by physicians in OM hospitals and a higher rate of western medication use in the national hospitals (54.5%) compared with that in the lower level hospitals (42.3%). Although the physicians in the national OM hospitals possessed more knowledge and tended to use more CAM therapies, their prescriptions may be further influenced by various marketing strategies of pharmaceutical companies, which do not reach their colleagues in lower level hospitals. The physicians who were trained as GPs in medical school tended to use more western medication than those who were trained as OMD, although the attitudes toward CAM therapies between these two groups were comparable. This finding suggests the need to enhance the knowledge of CAM and TM therapies of the GPs working in OM hospitals to better encourage more appropriately used integrative medicine.

The present findings must be interpreted in the context of the study's strengths and potential limitations. The strengths of this study were that participants were selected randomly after taking into account their geographic and hospital level factors, and the interviews were performed by a face-to-face method that resulted in a high response rate and less data collection bias. However, the participants who were physicians in the OM hospitals may not have represented the CAM practitioners in all of Vietnam because they provided the highest level of CAM services. In Vietnam, CAM can be practiced by both licensed physicians and practitioners. Because the knowledge of CAM practitioners did not originate from medical school, but was inherited from their parents, a high rate of Vietnamese folk medicine may be used among this population. Another limitation of this study is that the self-administered questionnaire may generate biases due to the respondents' overestimation of their capacity and perception of the questions. Although a concrete definition was provided for each CAM modality, the respondents may have been inclined to perceive chiropractice as a technique of massage and dietary therapy as a part of OM. The Cronbach's *α* coefficient of attitudinal scale was relatively low that may be partly due to a small number of items included, whereas knowledge and practice scales exhibited acceptable internal consistency. However, these scales should be validated for further application.

In conclusion, this study indicated that OM rather than Vietnamese folk medicine and other forms of CAM is the most commonly used CAM modality by physicians in OM hospitals in Vietnam. A broad range of CAM modalities and an excessive proportion of western medication were employed in conjunction with OM in the physicians' daily practice. This trend was influenced by the source of the CAM knowledge obtained, the education level, the specialty of the physicians, and the work environment. These findings suggest the marked interest in other CAM therapies in addition to traditional modes among physicians in the OM hospitals in Vietnam.

## Figures and Tables

**Table 1:** Demographic characteristics of the respondents.

	Provincial hospital	National hospital	*P*	Total
Response rate *n*/total (%)	239/257 (93.0)	73/80 (91.3)	0.78	312/337 (92.6)
Age (yrs)				
>45yrs (%)	28.5	43.8		32.1
36–45 yrs (%)	34.7	34.2	0.02	34.6
≤35 (%)	36.8	21.9		33.3
Gender				
Men (%)	52.7	41.1	0.11	50.0
Women (%)	47.3	58.9		50.0
Department				
Inpatient (%)	79.5	64.4	0.008	76.0
Outpatient (%)	20.5	35.6		24.0
Number of patients seen per day (pts)				
≥21 pts (%)	31.4	13.7		27.2
11–20 pts (%)	45.6	28.8	<0.0001	41.7
≤10 pts (%)	23	57.5		31.1
Length of experience in TM practice (yrs)				
≥20 yrs (%)	17.2	45.2		23.7
10–19 yrs (%)	38.5	32.9	<0.0001	37.2
<10 yrs (%)	44.4	21.9		39.1
Education in TM				
Undergraduate (%)	57.3	38.4	0.004	52.9
Graduate (%)	42.7	61.6		47.1
Running private clinic				
Yes (%)	25.9	20.5	0.35	24.7
No (%)	74.1	79.5		75.3
Medical specialty				
GP (%)	24.7	38.4	0.02	27.9
OMD (%)	75.3	61.6		72.1

*P* was calculated by chi-squared tests. TM: traditional medicine; GP: physicians who were trained as general practitioners in medical school; OMD: physicians who were trained as oriental medical doctors in medical school.

**Table 2:** Source of knowledge of CAM therapies among physicians in oriental medicine hospitals.

	Provincial hospital	National hospital	*P*	Total
College curriculum (%)	98.7	98.6	0.94	98.7
Text book (%)	82.0	90.4	0.09	84.0
Academic journal (%)	73.2	90.4	0.002	77.2
Internet (%)	69.9	86.3	0.005	73.7
Conference/training (%)	67.8	89.0	0.0004	72.8
Magazine/newspaper (%)	49.0	74.0	0.0002	54.8
Inherited from community (%)	24.7	42.5	0.003	28.8
Inherited from relatives (%)	26.8	31.5	0.43	27.9

*P* was calculated by chi-squared tests.

**Table 3:** Attitude toward CAM therapies among physicians in oriental medicine hospitals.

Questions	Absolutely agree	Agree	Disagree	Absolutely disagree
(Q1) Trust on effectiveness of CAM therapies.	75.0	25.0	0.0	0.0
(Q2) CAM may be better than conventional medicine because of its natural source.	51.9	42.0	6.1	0.0
(Q3) CAM emphasizes on holistic approach rather than disease.	40.1	34.9	22.1	2.9
(Q4) CAM may be superior to conventional medicine in some cases.	62.2	34.0	2.9	1.0
(Q5) CAM use should be based on scientific evidence.	35.6	43.9	18.9	1.6

Data are presented as percent of positive response.

**Table 4:** Association between demographic factors and overall scores of knowledge, practice, and attitude toward CAM and western medication use.

	Knowledge score		Practice score		Western medication use (%)		Attitude score	
	*β* (95% CI)	Mean (SE)^a^	*β* (95% CI)	Mean (SE)^a^	*β* (95% CI)	Mean (SE)^a^	*β* (95% CI)	Mean (SE)^a^
Age (yrs)								
>45 (*n* = 100)	1.97 (0.004 to 3.93)	26.2 (0.6)	−0.45 (−1.32 to 0.43)	5.5 (0.3)	−5.69 (−16.0 to 4.6)	45.8 (3.2)	0.45 (−0.22 to 1.12)	10.3 (0.2)
36–45 (*n* = 108)	0.77 (−0.87 to 2.42)	25.0 (0.6)	−0.33 (−1.07 to 0.40)	5.6 (0.3)	−3.58 (−12.2 to 5.05)	47.9 (3.1)	0.32 (−0.25 to 0.88)	10.1 (0.2)
≤35 (*n* = 104)	Ref	24.2 (0.8)	Ref	6.0 (0.3)	Ref	51.5 (4.0)	Ref	9.8 (0.3)
Gender								
Men (*n* = 156)	0.26 (−0.84 to 1.35)	25.2 (0.5)	0.08 (−0.41 to 0.57)	5.7 (0.2)	4.71 (−1.04 to 10.5)	50.8 (2.6)	0.21 (−0.16 to 0.59)	10.2 (0.2)
Women (*n* = 156)	Ref	25.0 (0.5)	Ref	5.7 (0.2)	Ref	46.1 (2.6)	Ref	10.0 (0.2)
Department								
Inpatient (*n* = 237)	0.84 (−0.42 to 2.11)	25.5 (0.4)	0.26 (−0.30 to 0.82)	5.8 (0.2)	3.48 (−3.15 to 10.1)	50.2 (2.2)	−0.14 (−0.58 to 0.29)	10.0 (0.1)
Outpatient (*n* = 75)	Ref	24.7 (0.6)	Ref	5.6 (0.3)	Ref	46.7 (3.2)	Ref	10.1 (0.2)
Length of experience in TM practice (yrs)								
≥20 (*n* = 74)	−3.23 (−5.24 to −1.21)**	23.3 (0.8)	−0.69 (−1.59 to 0.21)	5.4 (0.3)	−7.03 (−17.6 to 3.52)	44.1 (4.0)	−0.97 (−1.66 to −0.28)**	9.5 (0.3)
10–19 (*n* = 116)	−1.14 (−2.63 to 0.34)	25.4 (0.6)	−0.39 (−0.13 to 1.05)	5.7 (0.3)	−1.18 (−8.97 to 6.62)	50.0 (3.0)	−0.38 (−0.89 to 0.13)	10.1 (0.2)
<10 (*n* = 122)	Ref	26.6 (0.6)	Ref	6.1 (0.3)	Ref	51.2 (3.4)	Ref	10.5 (0.2)
Number of patients seen per day (pts)								
≥21 (*n* = 85)	0.79 (−0.74 to 2.31)	25.8 (0.6)	0.65 (−0.03 to 1.34)	6.0 (0.3)	6.94 (−1.05 to 14.9)	55.3 (3.2)	−0.12 (−0.64 to 0.40)	10.0 (0.2)
11–20 (*n* = 130)	−0.66 (−1.97 to 0.66)	24.4 (0.6)	0.46 (−0.13 to 1.05)	5.8 (0.3)	−6.67 (−13.6 to 0.24)	41.7 (3.0)	0.07 (−0.38 to 0.52)	10.1 (0.2)
≤10 (*n* = 97)	Ref	25.1 (0.6)	Ref	5.3 (0.3)	Ref	48.3 (3.0)	Ref	10.1 (0.2)
Running private clinic								
Yes (*n* = 77)	0.78 (−0.74 to 2.31)	25.5 (0.6)	0.56 (−0.04 to 1.15)	6.0 (0.3)	1.88 (−5.16 to 8.92)	49.4 (3.3)	0.35 (−0.12 to 0.81)	10.2 (0.2)
No (*n* = 235)	Ref	24.7 (0.4)	Ref	5.4 (0.2)	Ref	47.5 (2.2)	Ref	9.9 (0.1)
Education in TM								
Graduate (*n* = 147)	2.75 (4.12 to 15.4)***	26.5 (0.5)	1.12 (0.51 to 1.74)***	6.3 (0.3)	−3.54 (−10.7 to 3.64)	46.6 (2.7)	0.79 (0.32 to 1.26)**	10.5 (0.2)
Undergraduate (*n* = 165)	Ref	23.7 (0.6)	Ref	5.1 (0.3)	Ref	50.2 (3.0)	Ref	9.7 (0.2)
Medical specialty								
OMD (*n* = 225)	1.48 (0.24 to2.72)*	25.8 (0.4)	−0.10 (−0.65 to 0.46)	5.6 (0.2)	−13.3 (−19.8 to −6.7)***	41.8 (2.3)	−0.36 (−0.78 to 0.07)	9.9 (0.1)
GP (*n* = 87)	Ref	24.4 (0.6)	Ref	5.7 (0.3)	Ref	55.0 (3.1)	Ref	10.2 (0.2)
Hospital level								
National (*n* = 73)	2.77 (1.36 to 4.18)***	26.5 (0.6)	1.06 (0.43 to 1.69)**	6.2 (0.3)	12.2 (4.86 to 19.6)**	54.5 (3.3)	0.07 (−0.41 to 0.55)	10.1 (0.2)
Provincial (*n* = 239)	Ref	23.7 (0.5)	Ref	5.2 (0.2)	Ref	42.3 (2.4)	Ref	10.0 (0.2)

Linear regression analysis in which age, gender, department, length of experience in TM practice (yrs), number of patients seen per day, experience running a private clinic, education in TM, specialty type, and hospital level were considered predictive factors. *β* (95% CI) beta coefficient and 95% confident interval; ^a^adjusted mean and standard error. Ref: the group was set up as reference. *P < 0.05;  **P < 0.01;  ***P < 0.001.
